# Dynamical Mapping of *Anopheles darlingi* Densities in a Residual Malaria Transmission Area of French Guiana by Using Remote Sensing and Meteorological Data

**DOI:** 10.1371/journal.pone.0164685

**Published:** 2016-10-17

**Authors:** Antoine Adde, Emmanuel Roux, Morgan Mangeas, Nadine Dessay, Mathieu Nacher, Isabelle Dusfour, Romain Girod, Sébastien Briolant

**Affiliations:** 1 Unité d’Entomologie Médicale, Institut Pasteur de la Guyane, Cayenne, French Guiana; 2 UMR ESPACE-DEV, Institut de Recherche pour le Développement, Montpellier, France; 3 Centre d’Investigation Clinique et Epidémiologie Clinique Antilles Guyane, Centre hospitalier Andrée-Rosemon, Cayenne, French Guiana; 4 Direction Interarmées du Service de Santé en Guyane, Cayenne, French Guiana; 5 Unité de Parasitologie et d’Entomologie Médicale, Institut de Recherche Biomédicale des Armées, Marseille, France; 6 Unité de Recherche en Maladies Infectieuses Tropicales Emergentes, Faculté de Médecine La Timone, Marseille, France; Johns Hopkins University, UNITED STATES

## Abstract

Local variation in the density of *Anopheles* mosquitoes and the risk of exposure to bites are essential to explain the spatial and temporal heterogeneities in the transmission of malaria. Vector distribution is driven by environmental factors. Based on variables derived from satellite imagery and meteorological observations, this study aimed to dynamically model and map the densities of *Anopheles darlingi* in the municipality of Saint-Georges de l’Oyapock (French Guiana). Longitudinal sampling sessions of *An*. *darlingi* densities were conducted between September 2012 and October 2014. Landscape and meteorological data were collected and processed to extract a panel of variables that were potentially related to *An*. *darlingi* ecology. Based on these data, a robust methodology was formed to estimate a statistical predictive model of the spatial-temporal variations in the densities of *An*. *darlingi* in Saint-Georges de l’Oyapock. The final cross-validated model integrated two landscape variables—dense forest surface and built surface—together with four meteorological variables related to rainfall, evapotranspiration, and the minimal and maximal temperatures. Extrapolation of the model allowed the generation of predictive weekly maps of *An*. *darlingi* densities at a resolution of 10-m. Our results supported the use of satellite imagery and meteorological data to predict malaria vector densities. Such fine-scale modeling approach might be a useful tool for health authorities to plan control strategies and social communication in a cost-effective, targeted, and timely manner.

## Introduction

Nearly half of the world’s population, i.e., 3.2 billion people, is exposed to the risk of malaria [1]. Malaria is caused by *Plasmodium* parasites transmitted to humans through the bite of infected *Anopheles* mosquitoes. At every level of endemicity, the spatial and temporal distributions of malaria transmission are heterogeneous [2]. Local variation in abundance and exposure to the vectors is the key to explaining this heterogeneity [3, 4]; the more individuals that are bitten, the more likely are they to become infected and enhance transmission by infecting new mosquitoes. Accurate information on where and when malaria vectors proliferate is essential for malaria surveillance and elimination since it allows targeted interventions that remarkably increase the efficiency of control measures [5].

Malaria vector distribution is strongly influenced by environmental factors that determine the availability and productivity of *Anopheles* habitats. Environmental factors can be accurately detected and spatially analyzed using remote sensing techniques to characterize *Anopheles* ecological preferences, model population densities, and produce hazard maps [6–9]. The visual nature of maps makes them helpful tools to identify locations where interventions can be targeted [10]. Demand for maps of worldwide malaria-vulnerable areas, e.g., to steer World Health Organization (WHO) international actions, has led to an increasing number of studies aiming to circumscribe the geographical distribution of vectors [11–16]. Global maps from these studies constitute intelligent support, depicting the schematic large-scale distribution of the species. However, the spatial resolution of current maps is too coarse to capture the local spatial and temporal dynamics of *Anopheles*, which are very heterogeneous, even at fine scales [17–19]. The high heterogeneity of the *Anopheles* distribution, combined with major knowledge gaps in anopheline bio-ecology and experience from previous control efforts, suggest that ecological studies and control policies should be tailored to individual areas [20].

French Guiana is an overseas French territory of 230,000 inhabitants located in northern South America. Despite a continual decrease in the annual cases over the past decade (1.8 cases per 1,000 inhabitants in 2015), malaria remains a public health issue [21, 22]. *Plasmodium vivax* is the predominant malaria parasite species and was responsible for 80% of the diagnosed cases in 2015, with the remainder mainly due to *P*. *falciparum* [21–24]. Most malaria cases are reported in villages located along the main rivers flowing through the territory and in illegal gold mining areas, which are propitious places for malaria transmission [23–25]. As in a large part of the Americas, the main malaria vector in French Guiana has historically been considered to be *An*. *darlingi* [26–32], from its natural infectivity, anthropophilic behavior, high density during malaria transmission periods, and wide distribution [33–37]. Although *An*. *darlingi* has been the target of numerous studies, many aspects of its ecology and biology are still unknown. This species is highly adaptable, behaviorally variable, and has been observed in heterogeneous breeding sites in a wide range of ecosystems, including forest, savannah, swamps, and human-disturbed environments [12, 26, 34, 36, 38–42].

This study was conducted in Saint-Georges de l’Oyapock, a persistent malaria-endemic municipality of French Guiana where *An*. *darlingi* have been found infected by *P*. *vivax* [43]. *Anopheles darlingi* densities were longitudinally monitored during the malaria transmission period (i.e., the September–November dry season) in 2012, 2013, and 2014 at eight different sites. In parallel, appropriate satellite imagery and meteorological data were selected and processed in order to extract a panel of variables potentially related to *An*. *darlingi* ecology. Based on these data, this study aimed to dynamically model and map densities of *An*. *darlingi* in the municipality of Saint-Georges de l’Oyapock at weekly intervals during the malaria transmission period. Models and density maps of *An*. *darlingi* contribute toward controlling malaria transmission by bridging the knowledge gaps in *An*. *darlingi* bio-ecology and providing tools for health authorities to effectively apply vector controls.

## Materials and Methods

### Geography of the study area

Saint-Georges de l’Oyapock (3.89° N, 51.8° W) is a municipality of French Guiana located in the Amazon forest at the eastern border with Brazil along the Oyapock River. The 3,855 inhabitants (data from the French Institute of Statistics and Economic Studies, 2012) are mainly concentrated in the city center and more sporadically in the peripheral Amerindian villages. Contrasts between neighborhoods can be important because the population stems from a great diversity of ethnic groups. Therefore, local habits such as agricultural practices or housing types can vary widely from one area to another. In the “traditional” Amerindian places, wooden houses are mostly built on stilts, and fishing or slash-and-burn agriculture is the main livelihood activities of the populations. In the “modern” places, populations have adopted a western lifestyle. Houses are built with solid materials (concrete, bricks, etc.) and are surrounded by planted and tended gardens. Landscape of the study area is characterized by four main elements—the forest, savanna, urban, and river. The eastern side of the city is bordered by the Oyapock River, and the western side is open to the savanna. The northern and southern boundaries of the town are delimited by dense primary forest. The climate is equatorial: hot, wet, and rainy. The average temperatures range between 26.05°C in February and 27.85°C in October. The mean annual cumulative rainfall is 3,345 mm, with four alternating seasons: a long, rainy season from April to June; a long, dry season from July to December; a short, rainy season from January to February; and a short, dry season in March.

### Mosquito collection

**Fig 1:**
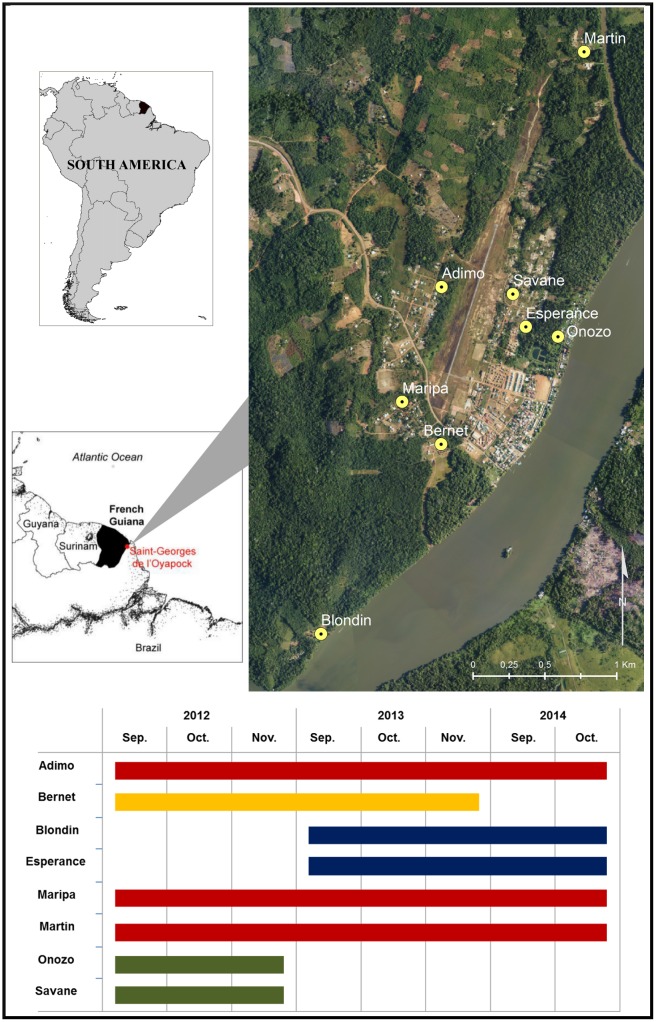
Study area and sampling sites. Location of collection sites with information about their sampling period, Saint-Georges de l’Oyapock, French Guiana. Aerial photograph acquired in 2006 (BD Ortho^®^ product from IGN, the French National Institute of Geographic and Forest Information).

Mosquitoes were collected during the dry seasons in 2012 (September 3–November 25; 12 weeks), 2013 (September 2–November 24; 12 weeks), and 2014 (September 1–October 26; 8 weeks) from eight different sites that varied by year (Fig 1). Sites were selected from field expertise and followed a longitudinal transect that maximized heterogeneity of the landscape and lifestyle characteristics of the population (Table 1). Landscape heterogeneities were decisive elements for sampling site selection. Photo interpretation and field expertise were used to select sampling sites such that the environmental richness of the study area was covered as best as possible. Since the lifestyle of the populations impacts the local environment (housing type or livelihood practices described in “Geography of the study area” section), this criterion was also considered. The eight sampling sites were finally decided by considering the best possible environmental criteria and field constraints (field hostility, access to the site, low risk of trapping material theft, etc.). Importantly, the choice of sampling sites was not oriented by the identification of places where we were confident to find numerous *An*. *darlingi*. Trapping sessions were conducted on two non-consecutive nights per week, between 6:00 pm and 8:00 am, by using Mosquito Magnet^®^ traps (Woodstream Corporation, Lititz, PA) baited with octenol (MMoct). Such traps have previously been proven useful to monitor the spatial and temporal abundance of malaria vectors in French Guiana [38, 44]. Mosquitoes were stored at -20°C until counting and morphological identification [31, 45, 46]. To achieve a weekly cartographic output and to smooth variation between collection nights, the two bi-weekly observations were averaged to approximate a daily number of specimens and then multiplied by seven to approximate a weekly number of specimens. In all, 165 weekly approximated *An*. *darlingi* density records across the eight study sites was finally available for analyses, which was slightly less than our expected maximum of 184, owing to the mechanical failure of traps. Since our activities were not conducted in a protected area (not a national park or a regional nature reserve), no specific permission was required, and field studies did not involve endangered or protected species.

**Table 1:** Characteristics of sampling sites.

Site	Landscape	Lifestyle
**Blondin**	River–Forest	Traditional
**Martin**	River–Forest–Savannah	Traditional
**Onozo**	River–Forest–Urban	Traditional
**Esperance**	Urban–Forest	Traditional
**Bernet**	Urban–Forest	Modern
**Maripa**	Savannah–Urban–Forest	Modern
**Adimo**	Savannah–Urban	Modern
**Savane**	Savannah–Urban	Traditional

Landscapes (photo interpretation and field expertise) and population lifestyles (field expertise) across the eight sites.

### Landscape characterization

**Fig 2:**
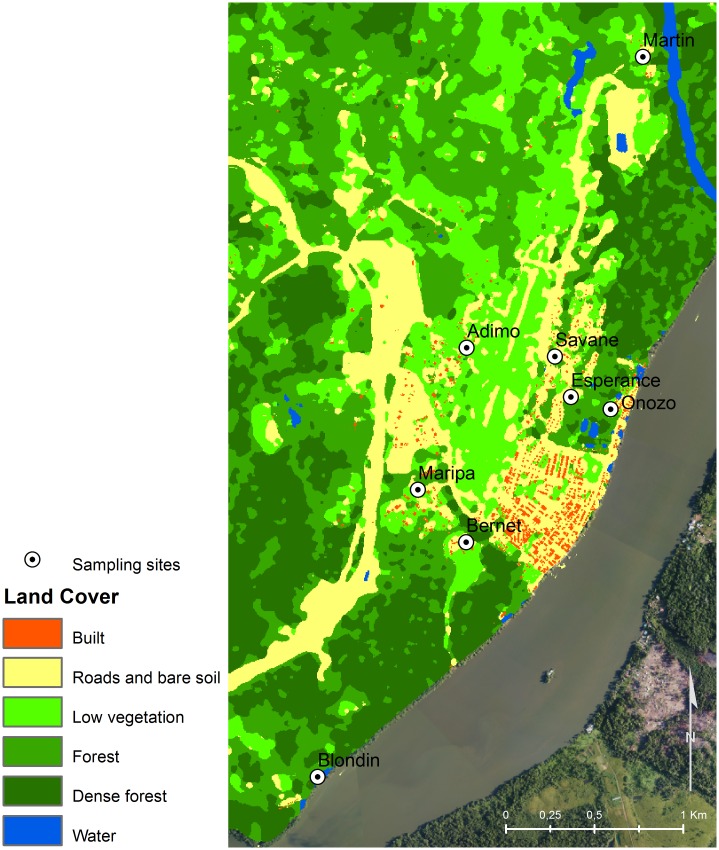
Land cover map of the study area, Saint-Georges de l’Oyapock, French Guiana.

A SPOT-5 image acquired on October 14, 2012, with four color channels (red, green, near-infra-red, and middle infrared) at 10-m spatial resolution was selected to characterize the landscape of the study area. One image was sufficient to cover the eight trapping sites. However, the presence of clouds required the posterior use of a second SPOT-5 image (July 22, 2013) in order to fill the missing data (5% of the total study area) and obtain a spatially complete product. A land cover map of the study area was produced based on field observations and a supervised training approach with maximum likelihood classification. The classification included five land cover types identified as “built, roads, and bare soils”; “low vegetation”; “forest”; “very dense forest”; and “water.” A 7 × 7 pixel mode filter (i.e., each pixel value being replaced by its most common neighbor in a 7 × 7 cell moving window) was applied to the classification to reduce noise. BD-Topo^®^ 2012 (IGN, the French National Institute of Geographic and Forest Information) was used to separate the “built” surfaces and “roads and bare soil” surfaces, resulting in a six-class land cover map (Fig 2). Land cover map quality was assessed by identifying the actual land cover of the training pixels by using photo-interpretation and local field expertise and applying a 5-fold cross validation procedure. The resulting mean Kappa coefficient was 0.84. A simplified binary classification, with two classes “forest” and “non-forest,” was also produced to provide complementary information for subsequent analyses. Biases from incomplete detection of water bodies, owing to forest canopy and thick vegetation covering the surface of water points were avoided by excluding the “water” land cover class from further statistical analyses. The remaining classes refer to perennial environmental facies. Therefore, changes in land cover during the study period were considered negligible, supporting the use of two satellite images acquired during different months to classify the study area. The Oyapock River and its main tributaries were excluded from the analysis since they are not a suitable habitat for *An*. *darlingi* breeding sites. Indeed, the estuarine situation of Saint-Georges de l’Oyapock implied a daily tidal influence and recurrent leaching of the riverbanks.

Given the lack of knowledge on the specific bio-ecology and behavior of *An*. *darlingi* in Saint-Georges de l’Oyapock, an exploratory approach was adopted by extracting a wide range of variables to characterize the landscape. These variables were extracted in a 200-m radius buffer around each trap by using FRAGSTATS software [47]. The radius represented a compromise between a relevant landscape characterization according to the satellite image spatial resolution, the overlap between neighboring buffers (to avoid information redundancy and artificial spatial auto-correlation), and the MMoct constructor attraction radius (up to 50 m). In total, 22 landscape variables were computed for each sampling site (Table 2). Four variables (PAFRAC, SPLIT, TE, and PRD) were computed for each of the two classifications (i.e., the final and simplified forest/non-forest binary classifications) by considering all classes together. Two variables (AREA and EDGE) were computed for each of the land cover classes (i.e., “built,” “roads and bare soils,” “low vegetation,” “forest,” “very dense forest,” “binary forest,” and “binary non-forest”).

**Table 2:** Landscape variables.

Landscape variables	Unit	Description
**Perimeter–Area Fractal Dimension (*PAFRAC*)**	None	*PAFRAC* reflects the shape complexity across a range of spatial scales (patch sizes). It equals two divided by the slope of the regression line between the logarithm of patch area (m^2^) and the logarithm of patch perimeter (m).
**Splitting Index (*SPLIT*)**	None	*SPLIT* is the number of patches with a constant patch size when the landscape is subdivided into S (value of the splitting index) patches. It equals total landscape area (m^2^) divided by the sum of the patch area (m^2^), summed across all patches.
**Patch Richness Density (*PRD*)**	Number per 100 hectares	*PRD* equals the number of different patch types within the landscape divided by the total landscape area (m^2^).
**Total Edge (*TE*)**	Meters	*TE* equals the sum of the lengths (m) of all edge segments in the landscape.
Total Class Area (*) (*AREA*)	Hectares	*AREA* equals the sum of the areas (m^2^) of all patches of the corresponding patch type, divided by 10,000 (to convert to hectares).
Edge Density (*) (*EDGE*)	Meters per hectares	*EDGE* equals the sum of the lengths (m) of all edge segments involving the corresponding patch type, divided by the total landscape area (m^2^), and multiplied by 10,000 (to convert to hectares).

Variables were extracted using FRAGSTATS in a 200-m radius buffer around each trap. Landscape metrics marked with an asterisk were computed separately for each individual land cover class. Remaining metrics were computed with all land cover classes together.

### Meteorological variables

Daily meteorological records, including rainfall, temperature, relative humidity, solar radiation, and evapotranspiration (ETP) for the years 2012–2014 were obtained from the Meteo-France weather station located in the city center of Saint-Georges de l’Oyapock. ETP was approximated using the Penman–Monteith equation [48]. For each meteorological parameter, a panel of variables (Table 3) was extracted using two different time interval schemes: a cumulative 7-day time interval scheme (i.e., past weather from days 0 to 6, days 0 to 13, days 0 to 20, etc.) and a non-cumulative 7-day time interval scheme (i.e., past weather from days 0 to 6, days 7 to 13, days 14 to 20, etc.). Extraction of meteorological variables ended at the ninth week (day 63) before the beginning of the trapping session to remain within the dry season. A total of 2,214 meteorological variables were extracted for each of the 32 weeks studied.

**Table 3:** Meteorological variables.

Raw data (unit)	Variables	Description
**Rainfall (mm) [Rain]**	*MaxNbConsecutiveDays_i-j_>PCTL*_*p*_	Maximum number of consecutive days that are above the *p*^th^ percentile, between days *i* and *j* before the trapping
**Temperature (°C) (minimal [TN], mean [TM], and maximal [TX])**	*MaxNbConsecutiveDays_i-j_<PCTL*_*p*_	Maximum number of consecutive days that are below the *p*^th^ percentile, between days *i* and *j* before the trapping
	*NbDays_i-j_>PCTL*_*p*_	Number of days that are above the *p*^th^ percentile, between days *i* and *j* before the trapping
**Relative Humidity (%) (min. [HN], mean [HM], and max. [HX])**	*NbDays_i-j_<PCTL*_*p*_	Number of days that are below the *p*^th^ percentile, between days *i* and *j* before the trapping
**Solar radiation (W/m²) [SR]**	*Max_i-j*	Maximal value between days *i* and *j* before the trapping
	*Min_i-j*	Minimal value between days *i* and *j* before the trapping
**Evapotranspiration (mm) [ETP]**	*Mean_i-j*	Mean value between days *i* and *j* before the trapping
**Rain (mm) [Rain]**	*MaxNbConsecutiveDaysNoRain_i-j*	Maximal number of consecutive days without rain between days *i* and *j* before the trapping
	*TotRain_i-j*	Cumulative rainfall between days *i* and *j* before the trapping
**Temperature (°C) (minimal [TN], mean [TM], and maximal [TX])**	*RangeTemp_i-j*	Difference between the maximal and minimal temperature values between days *i* and *j* before the trapping

Variables were extracted for two time interval schemes (i.e., cumulative 7-day and non-cumulative 7-day schemes). The 25^th^ and 75^th^ percentiles were used to extract temperature, relative humidity, solar radiation, and evapotranspiration. The 1^st^, 4^th^, 25^th^, 75^th^, 96^th^, and 99^th^ percentiles were used to extract rainfall.

### Statistical model

The spatial and temporal dynamics of *An*. *darlingi* densities in response to landscape and meteorology in Saint-Georges de l’Oyapock were assessed using a cumulative link mixed model (CLMM) [49]. The CLMM was estimated using maximum likelihood by using Laplace approximation with an ordinal response. Weekly *An*. *darlingi* densities were allocated to three classes following the tercile method: “Low densities” (first tercile), “Medium densities” (second tercile), and “High densities” (third tercile). The sampling scheme used potentially resulted in grouping structures as each trap and week providing several observations (replicates). In other words, *An*. *darlingi* densities observed in the same trap or in the same week were more likely to be similar than observations from different traps or weeks. CLMM allows multiple random effects to account for grouping variables and handling replications [50]. The ordinal response of *An*. *darlingi* density was analyzed using CLMM with logit link, two crossed random effects (trap position and catching week) and *C* = 3 categories (“Low,” “Medium,” and “High” densities):

$$logit\left(\gamma_{cjk}\right)=\alpha_{c}-\left(x_{jk}\prime \beta +u_{j}+\;u_{k}\right)\;\;\;\;\;\;\;c=1,2$$

where *k* = 1, 2, …, *n*_*k*_ is the week index and *j* = 1, 2, …, *n*_*j*_ is the trap, whereas *γ*_*cjk*_ is the cumulative probability up to *c*^th^ category for observation in trap *j* at week *k*. The covariate vector *x*_*jk*_includes the observation characteristic related to the regression coefficient (*β*), whereas the terms *u*_*j*_ and *u*_*k*_ are the random effects representing unobserved factors at the trap and week levels, respectively. The parameter *α*_*c*_ is the threshold (cutpoint). The probability of the category (*π*_*c*_) is obtained by the difference as follows:

$$\pi_{cjk}=\gamma_{cjk}-\gamma_{c-1,jk}=logit^{-1}\left(\alpha_{c}-\left(x_{jk}\prime \beta +u_{j}+u_{k}\right)\right)-logit^{-1}\left(\alpha_{c-1}-\left(x_{jk}\prime \beta +u_{j}+u_{k}\right)\right)$$

### Model selection

Three models were constructed. The spatial and temporal dynamics of *An*. *darlingi* densities were better and more easily evaluated by separately investigating the relative effects of the landscape and meteorological variables in the first and second models, respectively. Targeted landscape and meteorological variables were then merged into a third dynamic spatial-temporal model. Univariate CLMMs of *An*. *darlingi* densities were fitted using the 22 landscape and 2,214 meteorological features as explanatory variables. From the univariate analysis, landscape variables with *p*-values below 0.20 were retained for multivariate analyses. Because of the numerous meteorological variables, a different strategy was used: only variables listed in both the hundred lowest *p*-values and hundred highest log-likelihoods were retained for multivariate analyses. For collinear explanatory variables, we selected those that maximized the log-likelihood and were the most easily interpretable from a bio-ecological point of view. All possible multivariate combinations with the remaining variables were tested separately for landscape and meteorological features. Selection of the best spatial and temporal *An*. *darlingi* density models was based on statistical indicators, including minimization of the Akaike information criterion (AIC) [51], minimization of the random effects variance (RE), and maximization of the area under the curve (AUC) computed from a receiver operating characteristic (ROC) analysis [52]. Once defined separately, the landscape (spatial) and meteorological (temporal) variables were used together to fit a single spatial-temporal model. Weekly maps of *An*. *darlingi* densities in Saint-Georges de l’Oyapock were produced by calculating the probabilities of each pixel in the study area belonging to “Low,” “Medium,” or “High” classes according to the landscape and meteorological conditions for each of the 32 weeks of the study. For a given pixel, the class with the highest probability was assigned.

### Model validation

Model validity was assessed using a 10-fold cross-validation [53], which measures the stability of a model. The dataset was randomly partitioned into 10 parts, and the model was refitted 10 times with the partitions temporarily removed in rotation. Quality was evaluated by computing a cross-validated AUC (CVAUC), which was calculated as the average of the 10 AUC of the refitted models.

## Results

### *Anopheles darlingi* collections

Observed densities of *An*. *darlingi* in Saint-Georges de l’Oyapock showed high spatial and temporal heterogeneity (Table 4). The highest weekly mean densities were found in the peripheral Amerindian villages of Blondin and, to a lesser extent, in Martin, with 260.5 and 57.7 specimens, respectively. In the traps of Savane, Onozo, Adimo, Esperance, Maripa, and Bernet, near the city center, the weekly mean densities were lower, below 15 specimens. Temporal dynamics were characterized by maximal weekly densities observed in September (43.7) or October (69.0), depending on the study site. Minimal weekly densities were recorded at the end of the dry season in November (1.7). The spatial-temporal variation in *An*. *darlingi* densities is shown in detail in S1 Fig. The distribution of densities was characterized by a high number of null observations (Table 4). For example, the upper limit of the first tercile was 0 for the five traps in Adimo, Esperance, Maripa, Onozo, and Savane. Therefore, further modeling procedures used observations classified into three densities following the tercile method: “Low densities” ([0.0]), “Medium densities” ([0.0–12.2]), and “High densities” ([12.2–1,386.0]).

**Table 4:** *Anopheles darlingi* weekly density distribution.

Site	Mean				Median	1^st^ Tercile	2^nd^ Tercile	3^rd^ Tercile
	Sep.	Oct.	Nov.	Total				
**Adimo**	3.8	5.0	3.1	4.1	1.2	[0.0]	[0.0–3.5]	[3.5–21.0]
**Bernet**	23.7	12.5	2.0	12.2	3.5	[0.0–1.5]	[1.5–14.0]	[14.0–45.5]
**Blondin**	250.9	399.0	2.7	260.5	101.5	[0.0–37.7]	[37.7–159.2]	[159.2–1,386.0]
**Esperance**	6.8	11.7	0.0	7.7	0.0	[0.0]	[0.00–3.8]	[3.8–70.0]
**Maripa**	13.6	10.4	1.0	9.2	3.5	[0.0]	[0.0–7.0]	[7.0–56.0]
**Martin**	35.3	104.7	3.0	57.7	8.8	[0.0–2.0]	[2.0–39.1]	[39.1–378.0]
**Onozo**	8.2	0.9	0.0	3.0	0.0	[0.0]	[0.0–1.5]	[1.5–24.5]
**Savane**	1.8	1.8	0.0	1.2	0.0	[0.0]	[0.0]	[0.0–7.0]
**Total**	43.7	69.0	1.7	42.6	3.5	[0.0]	[0.0–12.2]	[12.2–1,386.0]

Sep, Oct, Nov: sampling months.

### Univariate analyses

Univariate predictive CLMMs of *An*. *darlingi* densities were fitted using each of the 22 landscape and 2,214 meteorological features as explanatory variables. Seven noncollinear landscape variables showed *p*-values lower than 0.20, and seven noncollinear meteorological variables were listed in both the hundred lowest *p*-values and the hundred highest log-likelihood values (Table 5). Following the model selection procedures, these variables were retained for multivariate analyses. For the landscape features, forest-related variables were positively associated, whereas all the other variables were negatively associated with *An*. *darlingi* densities. The selected meteorological variables included four related to temperature (T) and one each related to evapotranspiration (ETP), humidity (H), and rainfall (Rain). With the exception of “*TN_MaxNbConsecutiveDays_56–0_<22*.*5*” all the other variables were negatively associated with *An*. *darlingi* densities.

**Table 5:** Landscape and meteorological variables selected for multivariate CLMMs of *An*. *darlingi* densities during the malaria transmission period (i.e., September–November dry season) in Saint-Georges de l’Oyapock, French Guiana.

**Landscape variables**			
**Variable**	**Coefficient Sign**	**Log-likelihood**	***p*-value**
**[L1] *AREA_BareSoil***	-	-164.36	< 0.01
**[L2] *AREA_DenseForest***	+	-165.24	< 0.01
**[L3] *AREA_Built***	-	-164.73	< 0.01
**[L4] *PerimeterAreaFractalDimension***	-	-164.82	< 0.01
**[L5] *AREA_BinaryForest***	+	-167.29	0.01
**[L6] *EDGE_LowVegetation***	-	-166.48	0.03
**[L7] *TotalEdge***	-	-169.59	0.06
**Meteorological variables**			
**Variable**	**Coefficient Sign**	**Log-likelihood**	***p*-value**
**[M1] *ETP_max_28–0***	-	-158.61	< 0.01
**[M2] *HN_MaxNbConsecutiveDays_63–57_<49***	-	-159.41	< 0.01
**[M3] *MaxNbConsecutiveDaysNoRain_49–0***	-	-159.02	< 0.01
**[M4] *TM_moy_42–36***	-	-163.42	< 0.01
**[M5] *TN_MaxNbConsecutiveDays_56–0_<22*.*5***	+	-163.45	< 0.01
**[M6] *TX_moy_56–50***	-	-162.22	< 0.01
**[M7] *TX_MaxNbConsecutiveDays_63–57_>33*.*2***	-	-156.80	< 0.01

### Multivariate analyses

All possible multivariate combinations, separately for the landscape and meteorological variables listed in Table 5, were tested. Statistical performances of the significant predictive multivariate landscape- and meteorology-based models are listed in Table 6. It resulted in two landscape-based models and 14 meteorology-based models (only the five best models, i.e., with the highest explanatory value, are reported).

**Table 6:** Statistical performances of the best predictive multivariate landscape- and meteorology-based cumulative link mixed models of *An*. *darlingi* densities during the malaria transmission period (i.e., the September–November dry season) in Saint-Georges de l’Oyapock, French Guiana.

**Landscape-based models**						
**Model**	**Variables**	**AIC**	**AUC**			**RE**
			**Low**	**Medium**	**High**	
**LAND_1**	[L3] + [L4]	332.14	0.63	0.50	0.63	2.97
**LAND_2**	[L2] + [L3]	337.46	0.63	0.49	0.65	3.18
**Meteorology-based models**						
**Model**	**Variables**	**AIC**	**AUC**			**RE**
			**Low**	**Medium**	**High**	
**METEO_1**	[M1] + [M3] + [M5] + [M7]	297.11	0.68	0.54	0.71	1.91
**METEO_2**	[M1] + [M2] + [M3] + [M5]	298.72	0.68	0.56	0.71	1.90
**METEO_3**	[M1] + [M3] + [M5]	301.18	0.67	0.56	0.71	1.91
**METEO_4**	[M1] + [M5] + [M7]	304.55	0.71	0.55	0.70	1.25
**METEO_5**	[M1] + [M2] + [M5]	305.34	0.72	0.56	0.71	1.28

Landscape variable indices: “[L2]” for “*AREA_DenseForest*,” “[L3]” for “*AREA_Built*,” and “[L4]” for “*PerimeterAreaFractalDimension*.” Meteorological variables indices: “[M1]” for “*ETP_max_28–0*,” “[M2] for “*HN_MaxNbConsecutiveDays_63–57_<49*,” “[M3]” for “*MaxNbConsecutiveDaysNoRain_49–0*,” “[M5] for “*TN_MaxNbConsecutiveDays_56–0_<22*.*5*,” and “[M7]” for “*TX_MaxNbConsecutiveDays_63–57_>33*.*2*.” AIC: Akaike information criterion. AUC: area under the curve from the receiver operating characteristic (ROC) analysis for the Low, Medium, and High *An*. *darlingi* density classes. RE: Random effects total variance.

Selection of the final multivariate landscape- and meteorology-based CLMMs is detailed in S1 Text. For landscape, the LAND_2 model was selected. In this model, an increase in the built surface and dense forest surface in a 200-m radius around a trap was associated with a decrease and increase in *An*. *darlingi* densities, respectively. For meteorology, the METEO_1 model was selected showing the following: the maximal value of ETP during the 28 days preceding the collection (“*ETP_max_28–0*”) was negatively associated with *An*. *darlingi* densities; the maximum number of consecutive days without rainfall during the 49 days preceding the collection (“*MaxNbConsecutiveDaysNoRain_49–0*”) was negatively associated with *An*. *darlingi* densities; the maximum number of consecutive days with a minimal temperature below 22.5°C (25^th^ percentile) during the 56 days preceding the collection (“*TN_MaxNbConsecutiveDays_56–0_<22*.*5*”) was positively associated with *An*. *darlingi* densities; and the maximum number of consecutive days with a maximal temperature above 33.2°C (75^th^ percentile) during the ninth week preceding the collection (“*TX_MaxNbConsecutiveDays_63–57_>33*.*2*”) was negatively associated with *An*. *darlingi* densities.

### Spatial-temporal model

The targeted landscape and meteorological variables were used together to fit a single final spatial-temporal predictive model (Table 7). The AIC of the model was 287.05, and the AUC of the “Low,” “Medium,” and “High” *An*. *darlingi* density classes were 0.78, 0.64, and 0.80, respectively.

**Table 7:** Parameters of the predictive spatial-temporal cumulative link mixed model of *An*. *darlingi* densities during the malaria transmission period (i.e., the September–November dry season) in Saint-Georges de l’Oyapock, French Guiana.

	Coefficients	Standard errors	*P*-value
**Thresholds**			
** Low | Medium**	-22.69	5.34	
** Medium | High**	-20.73	5.29	
**Slopes**			
***AREA_BUILT***			
**By one hectare increase**	-3.67	1.41	< 0.01
***AREA_DENSFOREST***			
**By one hectare increase**	0.91	0.30	< 0.01
***ETP_max_28–0***			
**By one millimeter increase**	-3.65	0.93	< 0.01
***MaxNbConsecutiveDaysNoRain_49–0***			
**By one day increase**	-0.43	0.16	< 0.01
***TN_MaxNbConsecutiveDays_56–0_<22.5***			
**By one day increase**	0.10	0.03	< 0.01
***TX_MaxNbConsecutiveDays_63–57_>33.2***			
**By one day increase**	-0.22	0.09	0.01
**Random effects**			
** Trap**	0.14		
** Week**	< 0.01		

### Mapping *An*. *darlingi* densities

**Fig 3:**
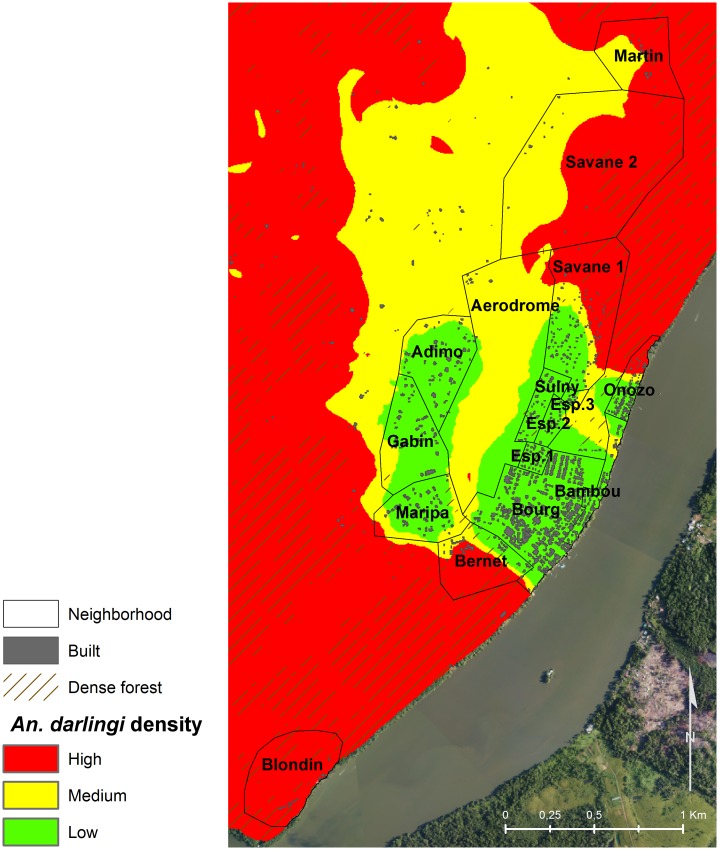
Landscape-based model map of predicted *An*. *darlingi* densities during the malaria transmission period (i.e., the September–November dry season) in Saint-Georges de l’Oyapock, French Guiana.

Extrapolation of the landscape-based model produced a static map of *An*. *darlingi* densities during the dry season (September–November) for the entire municipality of Saint-Georges de l’Oyapock (Fig 3), depicting the background influence of the landscape on *An*. *darlingi* densities, irrespective of intra-seasonal variation. In agreement with the model parameters, the densely forested surroundings of Saint-Georges de l’Oyapock were predicted as a high *An*. *darlingi* density area. This area encompassed the Amerindian villages of Blondin and Martin and included locations closer to the city center, such as the northern parts of the Onozo and Savane neighborhoods and a major part of the Bernet neighborhood, which is a military camp. The savannah plain in the northeast part of the city, mainly characterized by low vegetation (Fig 2), as well as the aerodrome neighborhood dominated by herbaceous vegetation, was predicted as medium *An*. *darlingi* density area. The most urbanized areas, i.e., city center neighborhoods, were predicted as low *An*. *darlingi* density areas.

Extrapolation of the spatial-temporal model (Table 7), based on both the land cover map of the study area and meteorological observations, allowed to dynamically map *An*. *darlingi* densities at weekly time intervals (S1 Movie). For the three sampling periods of 2012, 2013, and 2014, maps showed that the model predicted a global dynamic characterized by high *An*. *darlingi* densities in September across almost the entire study area, except most of the urbanized neighborhoods. Densities of *An*. *darlingi* then progressively decreased until the end of the dry season and maintained a spatial gradient from the city center neighborhoods (lowest densities) to the densely forested areas (highest densities).

### Model validation

AUC values were previously computed using the entire data set to guide model selection. The quality of the model was assessed by calculating a CVAUC from a 10-fold cross validation. The CVAUC means (standard deviations) were 0.78 (< 0.01), 0.64 (0.01), and 0.79 (0.02) for the “Low,” “Medium,” and “High” *An*. *darlingi* density classes, respectively. These values were very close to those computed using the entire data set (0.78, 0.64, and 0.80) and had low standard deviations. These results confirmed the stability of the model and its predictive value for the “Low” and “High” *An*. *darlingi* density classes.

## Discussion

Although *An*. *darlingi* is one of the most important malaria vectors in the Americas, in many areas, the influence of ecological factors on local population dynamics is not clearly understood. To our knowledge, this is the first study to implement a dynamic model of *An*. *darlingi* densities in French Guiana to assist the development of vector control strategies. An original and powerful modeling method was proposed based on a CLMM that exploits the ordered nature of observations and offers a flexible regression framework. The model selection procedures were guided by objective statistical criteria while retaining the central role of entomological expertise. The final model was based on two landscape variables (dense forest surface and built surface) and four meteorological variables (absence of rainfall, maximal ETP, and minimal and maximal temperatures). The accuracy of predicting the spatial-temporal densities of *An*. *darlingi* was evaluated by using a CVAUC, which was 0.74 in average for the three density classes. The average performance was reduced by the “Medium” density class, which had an AUC of 0.64; therefore, the predictions for intermediate density areas remain uncertain. More importantly, the best AUC value (0.80) was observed for the “High” density class. In other words, the model was the most accurate for the areas needing the highest malaria vector control.

Dense forest surface was an efficient predictor of high *An*. *darlingi* densities. However, the classification of the unpopulated dense forest surrounding Saint-Georges de l’Oyapock as a high *An*. *darlingi* density area should be discussed since this species is known for its anthropophilic behavior [36, 37, 54, 55]. Several specific characteristics of the study, combined with the ecological preferences of *An*. *darlingi*, might explain this model prediction. First, the hostility of the environment, i.e., the equatorial primary forest, prevented the placing of traps far from the edges of the dense forest; hence, all the sampling sites were effectively located close to human settlements. Thus, model predictions depicted that villages encompassing large patches of dense forest at their borders are highly exposed to malaria vectors. Second, the extrapolation of the model to the entire study area was not random. Indeed, the study was focused on a circumscribed area so that any pixel of the predictive map was not further than 2 km from human settlements, i.e., from *An*. *darlingi* blood meal sources. Although little is known about the flight range of *An*. *darlingi*, densely forested places in the study area could very less likely be the hotspots for *An*. *darlingi*. Forest ecosystems with human settlements at their borders are reported to provide optimal breeding, resting, and feeding sites that favor a high density and survival of human malaria vectors [9, 56].

Built surfaces were negatively associated with *An*. *darlingi* densities. Densely built-up areas favor runoff and are not likely to offer natural water bodies suitable for *An*. *darlingi* breeding or resting [26]. In addition, the increasing density of human settlement from the periphery of the municipality to the city center constitutes a behavioral barrier for mosquitoes. Females do not need to fly far into the city area to secure a blood meal. Similar observations and hypotheses have been made for *An*. *gambiae* in Africa with the number of specimens being inversely proportional to the level of urbanization in the area [57, 58].

A main limitation of this study is the absence of landscape variables directly related to the water bodies that are essential for breeding mosquitoes. This is an inherent flaw in the Amazonian region, which prevents the effective use of optical satellite imagery to exhaustively detect water bodies under the thick forest cover. The dense forest surface variable used to model *An*. *darlingi* densities might be considered as proxy for the presence of water. Indeed, the majority of the hydrographic network could likely be hidden by the canopy. Thus, although this research supports the use of optical satellite imagery to predict *An*. *darlingi* densities, it is limited in comparison with similar studies conducted in open landscapes, such as in Sahelian Africa [57] or Europe [59]. Further studies that evaluate the potential of radar imagery to detect water bodies under the canopy might help rectify this limitation.

Another limitation implied by the use of optic satellite imagery in the equatorial area was the scarcity of cloud-free image. Thus, the landscape of the study area was characterized based on a single time point land cover map. From the very regular presence of the authors in Saint-Georges de l’Oyapock for field collections, environmental changes during the entire study period were considered negligible. Despite this, fine-scale landscape modifications might have occurred between September 2012 and November 2014. Further, the landscape changes within a season (September–November) were not objectively evaluated. These two issues might result in incorrect predictions of local *An*. *darlingi* densities. However, since the land cover classes targeted in the present study refer to coarse environmental facies, the potential impacts of environmental modifications on the results were considered as minor.

The maximal value of ETP during the 28 days preceding the collection period was negatively associated with *An*. *darlingi* densities. Mosquitoes exposed to high ETP are potentially subjected to desiccation. Further, ETP might be considered a proxy for the loss of breeding sites. The maximum number of consecutive days without rainfall during the 49 days preceding the collection period was also negatively associated with *An*. *darlingi* densities. Long, dry spells during the proliferation season of *An*. *darlingi* (i.e., the dry season) most likely caused the reduction of aquatic breeding sites and interrupted population dynamics, leading to low densities in the following weeks. The maximum number of consecutive days with a minimal temperature below 22.5°C and a maximal temperature above 33.2°C were positively and negatively associated with *An*. *darlingi* densities, respectively. With regard to ETP, very high temperatures might cause mosquito desiccation and accelerate the loss of aquatic breeding sites. In contrast, lower temperatures appeared to benefit mosquito breeding. In the study area, minimal temperatures never fell below 20°C and were always suitable for *An*. *darlingi*. In addition, during the dry season, the lowest daily temperatures were most often recorded on rainy days. Therefore, the relationship between the maximum number of consecutive days with a minimal temperature below 22.5°C and *An*. *darlingi* densities might indicate the impact of rainy episodes during the dry season on mosquito density. Meteorological variables selected in the final model were related to the weather conditions observed several weeks before the sampling sessions (from four to nine weeks), which might exceed the lifespan of *An*. *darlingi* females. Therefore, the impact of meteorological factors on *An*. *darlingi* should be interpreted over several generations, i.e., adverse weather conditions during a given generation might impact subsequent ones.

By using entomological data sampled both in the dry and wet seasons, Adde et al. (unpublished data) observed a negative correlation between monthly rainfall and *An*. *darlingi* densities. Further, other studies [60, 61] have suggested that this negative relationship highlights the local seasonal framework conducive to high *An*. *darlingi* densities: the driest months of September–November. However, when focusing on the dry season and downsizing to a weekly scale, the relationship appears to be reversed: rainy events during the dry season are beneficial for the proliferation of *An*. *darlingi*. One hypothesis is that, during the wet season (April–June), the breeding sites are flooded and are regularly flushed, resulting in a drastic reduction in mosquito densities [36]. Dry conditions during the offseason (July–August) and at the onset of the dry season (September–mid October) might decrease the level of water bodies suitable for breeding, and flushing events become rarer, resulting in high densities of mosquitoes. Drying of the water bodies continues through the following weeks, and thus the availability of suitable breeding places becomes limited again toward the end of the dry season (end-October–November), resulting in a substantial reduction in *An*. *darlingi* densities.

Significant technical, human, and financial resources were required to achieve the considerable entomological collection in this study. The collection period was restricted to extend spatial sampling, which was not possible to continue throughout the year. This study focused on the middle of the dry season (September–November), which is the major period of malaria transmission and the season when *An*. *darlingi* is abundant. Unfortunately, this collection period did not allow the measurement of the increase in mosquito densities after the wet season: it begins in September when the densities are almost maximal. Given the hypothetical differentiated impact of rainfall on *An*. *darlingi* densities across seasons outlined above, the four different models (Dry season; Offseason 1; Wet season; Offseason 2) would be necessary to capture the dynamics over the entire year.

Mapping vector-borne disease determinants and entomological hazards is highlighted by the WHO as a central feature for efficient and integrated vector control management [62]. Hazard maps should be considered as predictive support that provide relevant information about where and when vector control interventions could be focused. This information is essential for the success of malaria elimination programs [63–67]. This study was conducted at a local level to provide operational results consistent with the intended scale of spatial intervention. Inevitably, this limits the generality and reproducibility of the results in other contexts. However, considering the high heterogeneity of malaria vector dynamics, focusing on local specificities is necessary to devise sustainable and effective control tools, which are not possible to implement from synoptic or large-scale approaches.

## Conclusions

The analysis of the relationships between landscape parameters derived from SPOT-5 satellite imagery, meteorological observations, and *An*. *darlingi* densities allowed developing a robust and operational methodology to dynamically map malaria vector density in Saint-Georges de l’Oyapock, an area of French Guiana where residual malaria transmission still exists. Such a fine-scale modeling approach fills the knowledge gap in local *An*. *darlingi* bio-ecology and provides a tool for health authorities to establish effective vector control. Production of near real-time *An*. *darlingi* density maps can be implemented by automating satellite and meteorological data processing to aid public health authorities in planning control strategies and preparing social communication in a cost-effective, targeted, and timely manner.

## Supporting Information

S1 Fig *Anopheles darlingi* weekly densities. Weekly densities of *An*. *darlingi* in the eight study sites according to their respective sampling periods. Black crosses represent the non-sampled weeks owing to the technical failure of the traps.
(TIF)

S1 Text Selection of the multivariate landscape and meteorological models of *Anopheles darlingi* densities during the malaria transmission period (i.e., the September–November dry season) in Saint-Georges de l’Oyapock, French Guiana. (DOCX)

S1 Movie Dynamic mapping of the predicted *Anopheles darlingi* densities during the malaria transmission period (i.e., the September–November dry season) of 2012, 2013, and 2014 in Saint-Georges de l’Oyapock, French Guiana. (WMV)
