# Supplementary figures and images for: Dynamical Mapping of Anopheles darlingi Densities in a Residual Malaria Transmission Area of French Guiana by Using Remote Sensing and Meteorological Data
Source: PLoS One. 2016 Oct 17;11(10):e0164685. doi: 10.1371/journal.pone.0164685 (PMC5066951; doi:10.1371/journal.pone.0164685)

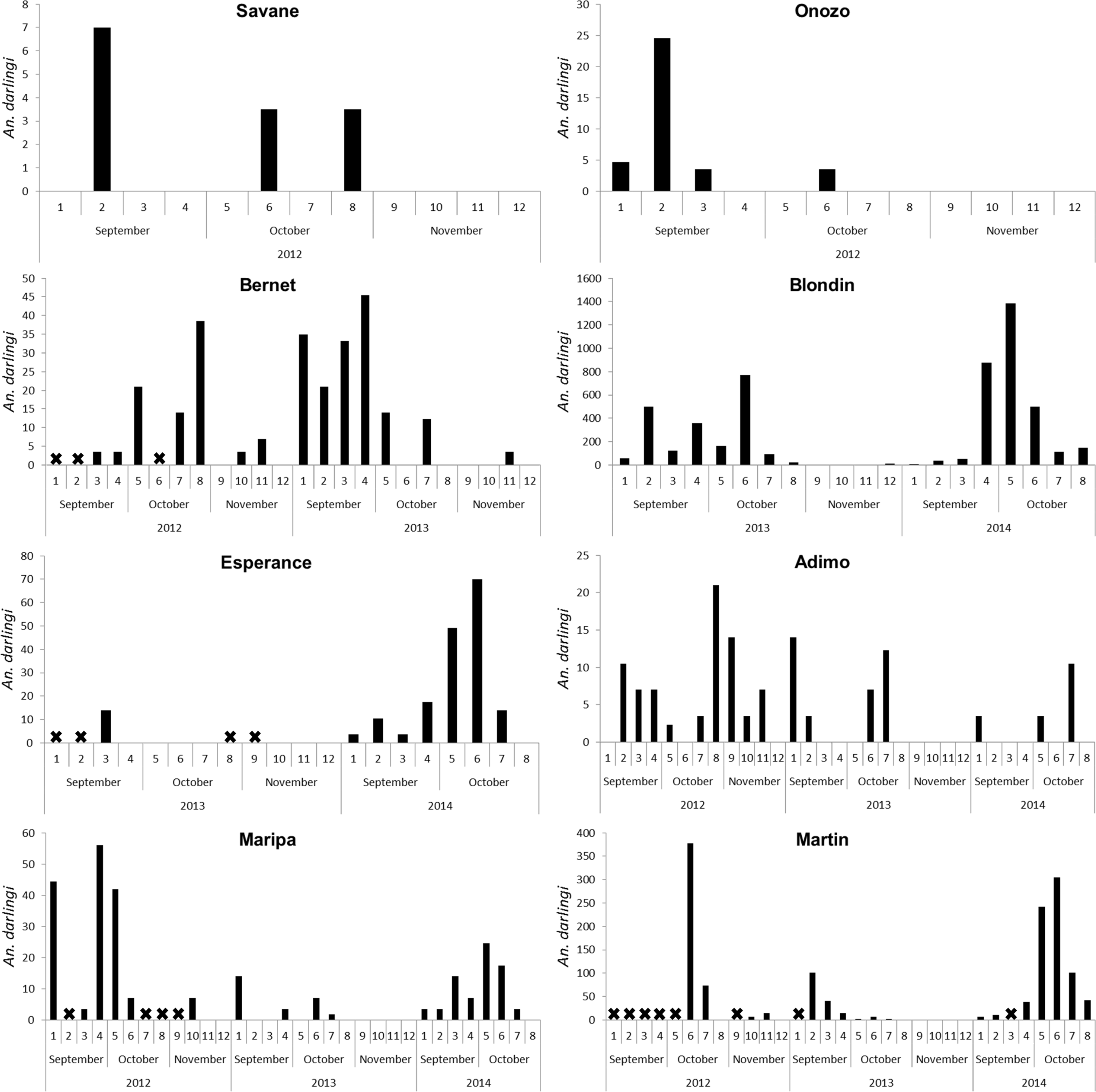

Supplement: S1 Fig — Black crosses represent the non-sampled weeks owing to the technical failure of the traps. (TIF) [file pone.0164685.s001.tif]
