# Supplementary material for: Dynamical Mapping of Anopheles darlingi Densities in a Residual Malaria Transmission Area of French Guiana by Using Remote Sensing and Meteorological Data
Source: PLoS One. 2016 Oct 17;11(10):e0164685. doi: 10.1371/journal.pone.0164685 (PMC5066951; doi:10.1371/journal.pone.0164685)
Supplement: S1 Text — (DOCX) [file pone.0164685.s002.docx]

1. **Landscape-based model selection**

The significant multivariate landscape-based models included two explanatory variables. The first (LAND_1) included built surface and PAFRAC index. The second (LAND_2) included built surface and dense forest surface. These two models showed close predictive properties. The AUC of the LAND_1 (LAND_2) model were of 0.63 (0.63), 0.50 (0.49) and 0.63 (0.65) for the low, medium and high *An. darlingi* density classes, respectively. The random effects variance (RE) was slightly minimized in LAND_1 (2.97 versus 3.18), as well as for the AIC (332.14 versus 337.46). Ultimately, given these similar performances, the choice of the final landscape-based model was done following entomological expertise. The integration of the *PAFRAC* variable in LAND_1 was criticized. *PAFRAC* reflects the complexity of the landscape and from the results of the present study a decreasing PAFRAC index was associated with an increasing of *An. darlingi* densities. This relationship was questionable in some cases. For example, low PAFRAC index, and as a result high *An. darlingi* densities were predicted in the city center of Saint-Georges de l’Oyapock. However, given the entomological expertise in the study area, this place is not subject to high *An. darlingi* densities. Conversely, the variables included in LAND_2 (built surface and dense forest surface) successfully passed the entomological expertise and were more easily interpretable from an ecological point of view. LAND_2 model was therefore selected (Table A).

**Table A. Parameters of the predictive landscape-based CLMM of *An. darlingi* densities during the malaria transmission period (i.e., the September–November dry season) in Saint-Georges de l’Oyapock, French Guiana.**

|  | Coefficients | Standard errors | P-value |
| --- | --- | --- | --- |
| Thresholds |  |  |  |
| Low \| Medium | -1.35 | 0.98 |  |
| Medium \| High | 0.53 | 0.97 |  |
| Slopes |  |  |  |
| *AREA_BUILT* |  |  |  |
| By one hectare increase | -4.06 | 1.73 | 0.02 |
| *AREA_DENSFOREST* |  |  |  |
| By one hectare increase | 0.82 | 0.36 | 0.02 |
| Random effects |  |  |  |
| Trap | 0.31 |  |  |
| Week | 2.87 |  |  |

1. **Meteorology-based model selection**

Among the five best meteorology-based models, two included four predictors and three included three predictors. As for the landscape-based models, they showed similar predictive values. The AUC were almost the same for the five models, with a maximum difference of 0.04 observed for the low *An. darlingi* densities class between METEO_5 and METEO_3. METEO_4 and METEO_5, both composed of three predictors, tended to minimize the RE (1.25 and 1.28, respectively). METEO_1 and METEO_2 were considered as well with high interest as they showed the lowest AIC values of 297.11 and 298.72, respectively, despite including four predictors. As the five models showed close statistical performances, the decision on the final meteorology-based model was highly oriented by entomological expertise. METEO_1 (Table B) was finally preferred because it had the advantage of including rainfall patterns explicitly. Moreover, METEO_1 showed the best AIC value despite including four predictors, discarding the overfitting hypothesis.

**Table B. Parameters of the predictive meteorology-based CLMM of *An. darlingi* densities during the malaria transmission period (i.e., the September–November dry season) in Saint-Georges de l’Oyapock, French Guiana.**

|  | Coefficients | Standard errors | P-value |
| --- | --- | --- | --- |
| Thresholds |  |  |  |
| Low \| Medium | -20.91 | 5.26 |  |
| Medium \| High | -18.94 | 5.20 |  |
| Slopes |  |  |  |
| *ETP_max_28-0* |  |  |  |
| By one mm increase | -3.41 | 0.92 | < 0.01 |
| *MaxNbConsecutiveDaysNoRain_49-0* |  |  |  |
| By one day increase | -0.47 | 0.16 | < 0.01 |
| *TN_MaxNbConsecutiveDays_56-0_<22.5* |  |  |  |
| By one day increase | 0.09 | 0.03 | <0.01 |
| *TX_MaxNbConsecutiveDays_63-57_>33.2* |  |  |  |
| By one day increase | -0.22 | 0.09 | 0.02 |
| Random effects |  |  |  |
| Trap | 1.91 |  |  |
| Week | < 0.01 |  |  |
